# Supplementary material for: Identification of individual root-knot nematodes using low coverage long-read sequencing
Source: PLoS One. 2021 Dec 1;16(12):e0253248. doi: 10.1371/journal.pone.0253248 (PMC8635404; doi:10.1371/journal.pone.0253248)
Supplement: S1 Appendix — (PDF) [file pone.0253248.s001.pdf]

# Supporting information

**Section 1: Reagent preparation**

Here we list all materials, reagents and equipment required and detail the preparation of stock and working solutions used in DNA extraction and library preparation.

**Section 2: SPRI based DNA extraction protocol**

An easy to follow protocol of the optimised Mu-DNA extraction method used in this project.

**Section 3: Library preparation**

Details of library preparation and the options available for measuring library concentration.

**Section 4: DNA extraction tests**

Although we used an optimised Mu-DNA extraction method in the project, this was compared to a Mu-DNA-phenol:chloroform method. Here we consider the performance and ease of use of each method.

**Section 5: Library compositions**

Samples used for each library are shown with sample id, species name, sample DNA concentration and total yield. Sequencing barcodes used in library construction and SRA accession are also given for each sample.

**Section 6: Database cleanup**

A summary of the RKN reference genomes used for the Kraken 2 database and the contaminants removed during cleaning.

**Section 7: Method time and cost**

Here we calculate the time and cost efficiency of our method.

# Section 1: Reagent preparation

## Materials

Sera-Mag SpeedBead Carboxylate-Modified Magnetic Particles (Hydrophobic), 15 mL (GE Healthcare: 65152105050250)
Rapid PCR Barcoding Kit (Oxford Nanopore: SQK-RPB004)
LongAmp Taq 2X Master Mix (NEB: M0287)
Qubit High-sensitivity (HS) dsDNA assay (Thermo Fisher Scientific: Q32851)

## Chemicals

5M HCl (Fisher Scientific: 10695872)
Tris HCl (Alfa Aesar: J67233.22)
Disodium EDTA dihydrate (Sigma Aldrich: E5134-250G)
Trisodium phosphate dodecahydrate (Sigma Aldrich: 04277-1KG)
Sodium chloride (Sigma Aldrich: S7653-250G)
Sodium dodecyl sulphate (Alfa Aesar: J75819.22)
Proteinase K (Thermo Fisher Scientific: AM2542)
Ammonium acetate (Sigma Aldrich: A1542-500G)
Aluminium ammonium sulphate dodecahydrate (Alfa Aesar: 13802.22)
Calcium chloride dihydrate (Sigma Aldrich: 1.02382.0250)

## Plastics

1.5 ml Eppendorf DNA LoBind Tubes (Fisher Scientific: 10051232)
10, 100, 200 and 1000 µl pipette tips (brand of choice)

## Equipment

Hula mixer (Thermo Fisher Scientific: 15920D) or similar
Eppendorf ThermoMixer C (Eppendorf: 5382000031) or similar
Magnetic racks for 1.5 ml tubes and 0.2 ml PCR tubes
Qubit 3.0 fluorometer (Fisher Scientific: 15387293) or similar

## Stock solutions

Stock solutions are given as compositions for 100 ml. Adjust as required.

***1 M Tris HCl (pH 8)***:
Add 15.7 g of Tris HCl to bottle. Add 75 ml ddH2O. Add 5 ml 5 M NaOH to adjust to pH 8. Bring to 100 ml final volume with ddH2O. Place on HulaMixer until all solids dissolve.

***0.5 M EDTA (pH 8)***:
Add 18.6 g of disodium EDTA dihydrate to bottle. Add 75 ml ddH2O and 10.2 ml 5 M NaOH to adjust to pH 8. Bring to 100 ml final volume with ddH2O. Place on HulaMixer until all solids dissolve.

***5 M Ammonium acetate***:
Add 38.6 g ammonium acetate to bottle. Bring to 100 ml final volume with ddH2O. Place on HulaMixer until all solids dissolve.

***180 mM Aluminium etc.***:
Add 8.2 g aluminium ammonium sulphate dodecahydrate to bottle. Bring to 100 ml final volume with ddH2O. Place on HulaMixer until all solids dissolve.

***3% Calcium chloride***:
Add 3 g calcium chloride dihydrate to bottle. Bring to 100 ml final volume with ddH2O. Place on HulaMixer until all solids dissolve.

***5 M NaCl*:**
Dissolve 29.2 g sodium chloride in 75 ml $ddH_2O$. Bring to 100 ml final volume with $ddH_20$.

***50% PEG 8000*:**
To 50 g polyethylene glycol 8000 add $ddH_20$ to a final volume of 100 ml. Place on HulaMixer until all solids dissolve.

***10% Tween 20*:**
To 900 ul $ddH_2O$ add 100 ul Tween 20. Invert repeatedly to mix.

**Note:** With the exception of ***10% Tween 20*** all stock solutions can be UV sterilised. Stock solutions can be stored at room temperature. Store ***10% Tween 20*** in the dark.

## Working solutions

***Lysis Solution***:
Add 8.7 g trisodium phosphate dodecahydrate and 0.2 g sodium chloride to bottle. Add 70 ml ddH2O, 6.7 ml ***1 M Tris HCl (pH 8)*** and 5.3 ml ***0.5 M EDTA (pH 8)***. Place on HulaMixer until all solids dissolve. Add 2.5 ml 5 M HCl to adjust to pH 9. Bring to 100 ml final volume with ddH2O. Invert to mix.

***Tissue Lysis Additive***:
To 600 ml (2 volumes) ddH2O add 300 ml (1 volume) ***20% SDS***. Invert to mix.

***Flocculant Solution*****:**
To 50 ml ***5 M Ammonium acetate*** add 25 ml ***180 mM Aluminium etc***. Invert to mix before adding 25 ml ***3% Calcium chloride***. Invert to mix.

***Wash Solution*****:**
To 80 ml 100% ethanol add 20 ml ddH2O. Invert to mix.

***Elution Buffer*****:**
To 1 ml ***1 M Tris HCl (pH 8)*** and 0.2 ml ***0.5 M EDTA (pH 8)***. Bring to 100 ml final volume with ddH2O. Invert to mix.

***Library Buffer*****:**
To 1 ml ***1 M Tris HCl (pH 8)*** and 1 ml ***5 M NaCl***. Bring to 100 ml final volume with ddH2O. Invert to mix.

***DNA Extraction Bead Solution (for final 10 ml vol)*****:**
Mix 100 ul ***1 M Tris HCl (pH 8)***, 20 ul ***0.5 M EDTA (pH 8)*** and 3.2 ml ***5 M NaCl***. Add 4 ml ***50% PEG 8000*** and invert to mix. Add 2.53 ml $ddH_20$. Invert to mix thoroughly. Add 50 ul of ***10% Tween 20*** then add 100ul prepared ***Bead suspension*** (see below). Place on HulaMixer until mixed thoroughly.

***Library Prep Bead Solution (for final 10 ml vol)*****:**
Mix 100 ul ***1 M Tris HCl (pH 8)***, 20 ul ***0.5 M EDTA (pH 8)*** and 3.2 ml ***5 M NaCl***. Add 4 ml ***50% PEG 8000*** and invert to mix. Add 2.43 ml $ddH_20$. Invert to mix thoroughly. Add 50 ul of ***10% Tween 20*** then add 200ul prepared ***Bead suspension*** (see below). Place on HulaMixer until mixed thoroughly.

**Note:** Working solutions can be stored at room temperature. Bead solutions should be refrigerated, avoid prolonged exposure to light. For best results, bead solutions should be replaced after 1 month.

### Bead suspension

It is simplest to take aliquots of beads from the Sera Mag SpeedBead bottle at adequate amounts for use, e.g. 100 ul for 10 ml ***DNA Extraction Bead Solution***. Allow Sera-Mag SpeedBeads bottle to reach room temperature. Vortex the bottle until the beads are completely resuspended - this may take some time but it is essential they are fully resuspended (i.e. they are not clumped on the base of the bottle). Immediately after resuspension transfer the desired volume of Sera-Mag SpeedBeads to a 1.5 ml tube. Store aliquots in the fridge ready for preparation.

***Bead suspension*** preparation:

1. Allow Sera-Mag SpeedBeads aliquot to reach room temperature.
2. Vortex thoroughly to resuspend beads. Centrifuge briefly to remove droplets from tube lid.
3. Place on magnetic stand until supernatant is completely clear and beads are bound towards magnet. This should take approximately ten minutes but can take longer.
4. While on the stand carefully remove and discard supernatant without disturbing beads.
5. Add 500 ul dd$H_2O$. Vortex tube to resuspend beads. Centrifuge briefly to remove droplets from tube lid.
6. Place on magnetic stand until supernatant is completely clear and beads are bound towards magnet. This should take approximately ten minutes but can take longer.
7. While on the stand carefully remove and discard supernatant without disturbing beads.
8. Repeat steps 5 to 7 three more times.
9. Add ***Elution Buffer*** to match the starting volume of aliquot. Vortex tube to resuspend beads. Centrifuge briefly to remove droplets from tube lid.
10. ***Bead suspension*** can now be added to the bead solution

**Note:** For steps 5 to 7 the amount of dd$H_2O$ added needs to be more than the starting volume of bead aliquot. If preparing 500 ul of beads adding 750 ul dd$H_2O$ is adequate.

# Section 2: SPRI based DNA extraction protocol

Thaw samples on ice. Centrifuge 1.5 ml Eppendorf LoBind tube containing nematode sample at ≥ 10,000 xg for 1 min to ensure sample is at bottom of tube. Pipette off excess water if sample is stored in water, be careful not to remove sample. A small volume of water remaining (~10 µl) will not affect extraction. Take ***DNA Extraction Bead Solution*** from the fridge and allow it to reach room temperature. Vortex to mix thoroughly.

### Lysis

1. Create ***Lysis Master Mix***: 730 µl ***Lysis Solution***, 250 µl ***Tissue Lysis Additive*** and 60 µl ***PK***. Vortex to mix
2. Add 200 µl ***Lysis Master Mix*** **to** 1.5 ml LoBind tube containing nematode sample. Vortex briefly to mix and centrifuge tube for 1 sec
3. Place in Thermomixer at 55°C for 90 mins at 550 rpm.
4. Centrifuge tube for 1 sec

### Inhibitor removal

5. Add 70 µl (0.3 X volume) of ***Flocculant Solution***, invert several times to mix and incubate on ice for a minimum of 10 min
6. Centrifuge at ≥ 10,000 xg for 2 min at room temperature
7. Without disturbing the pellet, transfer supernatant to a fresh 1.5 ml LoBind tube

### SPRI DNA binding

8. Add 400 µl (2 X volume) of ***DNA Extraction Bead Solution***. Place on HulaMixer (continual rotation) for 10 min
9. Place on magnetic stand until supernatant is clear and beads are bound towards magnet
10. While on the stand carefully remove and discard supernatant without disturbing beads

### Wash

11. Add 1000 µl ***Wash Solution***. Incubate at room temperature for 30 secs
12. While on the stand carefully remove and discard supernatant without disturbing beads
13. Repeat steps 11 to 12 a further time
14. Centrifuge tube for 1 sec. Place back on magnetic stand ensuring beads are bound towards magnet. Remove all remaining ***Wash Solution*** with a 10 µl pipette. Air dry tube with cap open until beads are completely dry (i.e. no longer shiny)

### Elution

15. Add 25 µl ***Elution Buffer*** (55°C) and gently flick to resuspend beads. Ensure all beads are resuspended with no clumps. Centrifuge tube for 1 sec
16. Place in Thermomixer at 55°C for 10 mins at 550 rpm. Centrifuge tube for 1 sec
17. Place on magnetic stand until supernatant is clear and beads are bound towards magnet
18. Carefully transfer eluate to a fresh 1.5 ml LoBind tube without disturbing beads

# Section 3: Library preparation and sequencing

This is a modified version of Oxford Nanopore Technologies' Rapid PCR Barcoding Kit (SQK-RPB004) protocol, optimised for RKN samples with extremely low input DNA for use on a Flongle.

## Preparation

Thaw samples on ice. Thaw Rapid PCR Barcoding Kit reagents: Barcodes (***RLB*** 01-12a) at room temperature and place Fragmentation Mix (***FRM***) on ice, vortex briefly and spin down.

## DNA fragmentation and tagmenting

Quantify 2 µl of each sample on a Qubit 3.0 fluorometer using high-sensitivity (HS) dsDNA assay (Invitrogen). If sample DNA concentration is less than 1 ng/µl (or 'too low') use 5 µl of sample for fragmentation, otherwise use 3 µl.

Add appropriate sample volume to 1 µl ***FRM*** in a 0.2 ml PCR tube, close lid and flick gently to mix. Centrifuge briefly to collect droplets from lid. Place on ice until required.

In a thermal cycler, incubate PCR tube at 30°C for 1 minute, then at 80°C for 1 minute and finally hold at 4°C. DNA template is now tagmented, place on ice until required.

## PCR barcoding

Make a PCR master mix per sample (allow ~5% for pipetting error):
    20 µl Nuclease-free water
    25 µl LongAmp Taq 2X master mix

Add 45 µl PCR master mix to each tagmented sample and 1 µl of ***RLB*** (01-12a). Flick gently to mix, centrifuge briefly to collect droplets from lid. Place on ice until required.

If initial sample DNA template > 0.1 ng/µl, place sample in a thermal cycler with:
    3 mins @ 95°C, ***15*** X (15 secs @ 95°C, 15 secs @ 56°C, 6 mins @ 65°C),
    6 mins @ 65°C, hold at 4 °C
Else, place sample in a thermal cycler with:
    3 mins @ 95°C, ***20*** X (15 secs @ 95°C, 15 secs @ 56°C, 6 mins @ 65°C),
    6 mins @ 65°C, hold at 4 °C

**Note:** PCR barcoded samples can be stored in a fridge overnight or upto a week if required.

## Sample concentration

A magnetic stand or rack is required for this step. If it cannot accommodate PCR strip tubes, transfer each PCR barcoded sample to a 1.5 ml Eppendorf DNA LoBind tube. Take ***Library Prep Bead Solution*** from the fridge and allow it to reach room temperature. Vortex to mix thoroughly. Incubate ***Library Buffer*** at 55°C until required.

1. Add 50 µl (1 X volume) of ***Library Prep Bead Solution*** to each sample. Mix by pipetting
2. Leave to stand at room temperature for 10 mins
3. Place on magnetic stand until supernatant is clear and beads are bound towards magnet
4. While on the stand carefully remove and discard supernatant without disturbing beads
5. Add 150 µl ***Wash Solution***. Incubate at room temperature for 30 secs
6. While on the stand carefully remove and discard supernatant without disturbing beads
7. Repeat steps 11 to 12 a further time
8. Centrifuge tube for 1 sec. Place back on magnetic stand ensuring beads are bound towards magnet. Remove all remaining ***Wash Solution*** with a 10 µl pipette. Air dry tube with cap open until beads are completely dry (i.e. no longer shiny)
9. Add 15 µl ***Library Buffer*** (55°C) and gently flick to resuspend beads. Ensure all beads are resuspended with no clumps. Centrifuge tube for 1 sec
10. Leave to stand at room temperature for 10 mins
11. Place on magnetic stand until supernatant is clear and beads are bound towards magnet
12. Carefully transfer eluate to a fresh 0.2 ml PCR tube without disturbing beads

**Note:** Concentrated samples can be stored in a fridge overnight or upto a week if required

## Sample DNA quantification

Quantify 2 µl of each concentrated sample on a Qubit 3.0 fluorometer using high-sensitivity (HS) dsDNA assay (Invitrogen).

## Sample DNA fragment length estimation

For sample DNA fragment length estimates there are three options we have used:

**Option 1** (ideal and accurate but expensive, does not work with low DNA concentration):
1 µl of each sample was measured on a Tapestation 2200 (Agilent Technologies) using genomic tape.

**Option 2** (cheap but less accurate, may not work with low DNA concentration):
2 µl of each sample was run on a 1% agarose gel against 0.5 µl Generuler 1 Kb DNA ladder (or similar, diluted 1:20). Estimate fragment length based on gel imaging.

**Option 3** (free but less accurate, DNA concentration not an issue):
Assume all samples have a fragment length of 7 Kb.

## Sample molarity estimation

Based on the sample quantification and fragment length estimates (whichever option chosen), the molarity of each sample can now be calculated in fmol/µl. For this use the following equation:

*fmol/µl = DNA concentration / ((DNA fragment length X 1,000 X 617.96) + 36.04) X 1,000,000*

**Note:** DNA concentration is in ng/µl, DNA fragment length is in Kb.

## Pool samples

Samples should be pooled as equimolar as possible for best results. However, this is not entirely necessary or sometimes even possible. Aim for a final library concentration of ~ 2 fmol/µl. This will mean that the 5 µl volume of library used for sequencing a full 12 samples will contain 5 - 10 fmol of DNA, ideal for Flongle sequencing.

It is perfectly feasible to concentrate (see sample concentration above) or dilute (using ***Library Buffer***) the pooled library as required. After adjusting the concentration it is advised to quantify 2 µl of the pooled library on a Qubit 3.0 and recalculate molarity (as above) using the average fragment length.

## Sequencing adapter ligation

Allow Rapid Adapter (***RAP***) to reach room temperature. Vortex briefly and spin down.

1. Transfer 5 µl of final library into a 1.5 ml Eppendorf DNA LoBind tube
2. Add 0.5 µl ***RAP*** to the tube. Flick gently to mix and spin down briefly
3. Incubate for 5 - 10 minutes at room temperature
4. Place on ice until required

## Loading the Flongle flow cell

**Note:** it is important to perform a flow cell check prior to loading to ascertain the flow cell's quality.

Follow any Flongle protocol from 'Loading the Flongle flow cell' (e.g. this protocol).

## Sequencing

We recommend specifying a directory for sequencing output (i.e. a desktop or external hard drive directory) rather than the hard-to-find default output directory of ONT's MinKNOW software. Disable all base calling options - we basecall the output fast5s with Guppy GPU HAC post sequencing. Run the sequencing for the default 24 hours. A longer sequencing period may allow for more data, however, we have found reads begin to plateau around the 24 hour period using our method.

# Section 4: DNA extraction method comparisons

### Mu-DNA-beads vs a Mu-DNA-phenol:chloroform-beads

Phenol:chloroform is a gold standard for DNA extraction. Here we test the efficiency of the Mu-DNA-beads vs a Mu-DNA-phenol:chloroform-beads extraction method. For both methods the Mu-DNA lysis step of the DNA extraction protocol was used.

### Mu-DNA-phenol:chloroform-beads method

Post lysis, in a well ventilated fume hood, add 1 X volume phenol:chloroform:isoamyl alcohol (25:24:1, v/v) to lysate. Invert to mix thoroughly. Centrifuge for 5 mins at ≥ 10,000 xg at room temperature. Transfer aqueous phase to fresh tube. Method then follows DNA extraction protocol from 'SPRI DNA binding' (no fume hood required).

### Comparison of methods

Samples tested:
C: cichlid fish, *Nimbochromis livingstonii*, flank tissue (~ 2 $mm^3$)
T: single tadpole shrimp, *Triops cancriformis*, eggs (~ 0.5 mm diameter)
M: single excised immature *M. incognita* females (~ 0.2 mm diameter)

For each sample type 4 replicates were processed using the same starting amount of tissue (visually size based). Replicates were crushed with a sterile tube pestle in 300 ul of ***Lysis Master Mix*** and incubated at 55°C for 2 hours. Post lysis, replicates of each sample type were pooled and mixed by vortexing. Pooled replicates were then split into 2 x 300 ul subsamples to be extracted by Mu-DNA-beads (M) or Mu-DNA-phenol:chloroform-beads (P). For example: cichlid tissue Mu-DNA-beads; CM. All samples were eluted in 50 ul ***Elution Buffer*** (200 ul for cichlid tissue). All samples were quantified with a Qubit 3.0 fluorometer high-sensitivity (HS) dsDNA assay (Invitrogen) and compared (Table S1).

**Table S1. Extraction method yields from various tissue samples.**
Sample types: cichlid tissue (C); single tadpole shrimp egg (T); single excised immature *M. incognita* female (M). Samples were extracted with either Mu-DNA-beads (M) or Mu-DNA-phenol:chloroform-beads (P). E.g. cichlid tissue extracted with Mu-DNA-beads: CM.

| Sample | Extraction method | Final volume (µl) | Total yield (ng) |
|---|---|---|---|
| CM | Mu-DNA/beads | 200 | Too high |
| TM | Mu-DNA/beads | 50 | 6.2 |
| MM | Mu-DNA/beads | 50 | 4.425 |
| CP | Mu-DNA/PCI/beads | 200 | Too high |
| TP | Mu-DNA/PCI/beads | 50 | 7.55 |
| MP | Mu-DNA/PCI/beads | 50 | 5.35 |

Cichlid tissue had too high a yield to quantify on the Qubit 3.0. Dilutions of 1:10 and 1:100 were quantified (Table S2).

**Table S2. Serial diluted samples of cichlid tissue from extraction method tests.** Cichlid tissue samples (C) extracted with either Mu-DNA-beads (M) or Mu-DNA-phenol:chloroform-beads (P). E.g. cichlid tissue extracted with Mu-DNA-beads: CM. Dilutions are shown as 1:10 or 1:100.

| Sample | Extraction method | Final volume (μl) | DNA conc (ng/μl) | DNA yield (ng) |
|---|---|---|---|---|
| CB 1:10 | Mu-DNA/beads | 2000 | 18.8 | 37600 |
| CB 1:100 | Mu-DNA/beads | 20000 | 1.71 | 34200 |
| CP 1:10 | Mu-DNA/PCI/beads | 2000 | 29.2 | 58400 |
| CP 1:100 | Mu-DNA/PCI/beads | 20000 | 2.43 | 48600 |

As the cichlid tissue samples performed so well the undiluted samples were analysed on a Nanodrop 1000 spectrophotometer (Thermo Fisher Scientific) to determine purity of DNA (Fig S1).

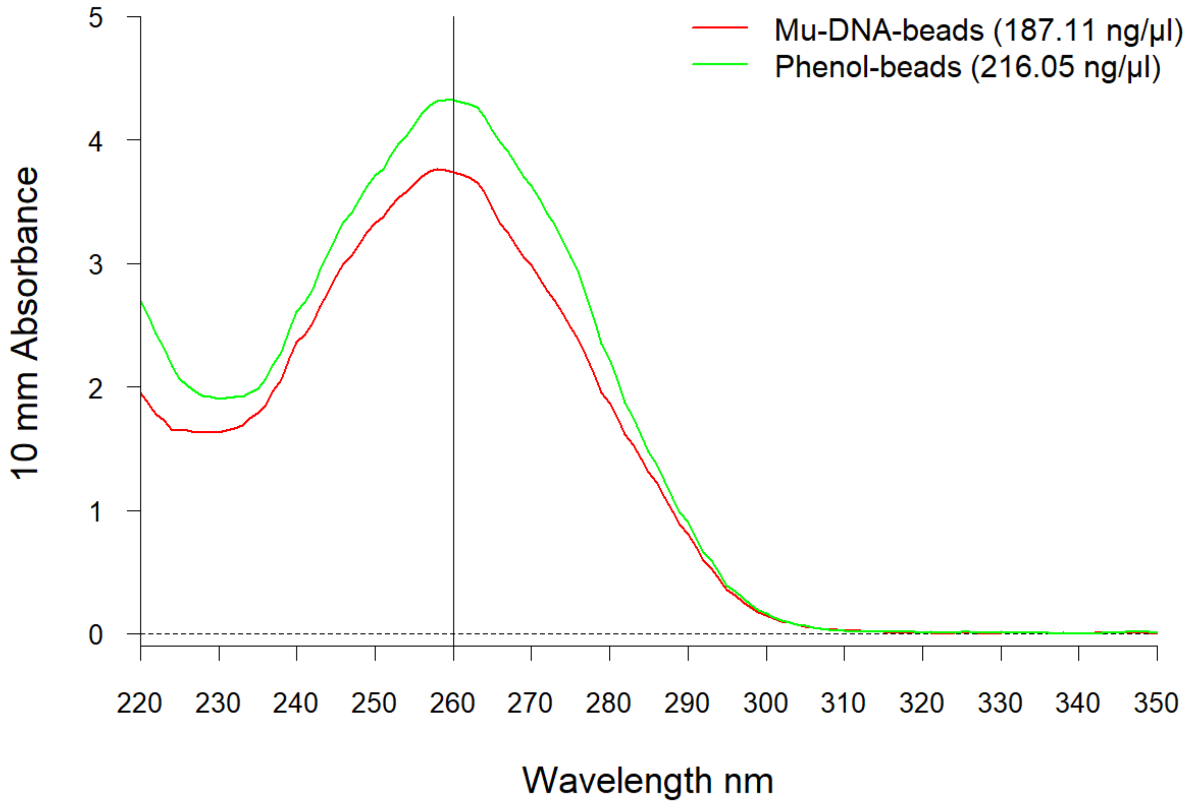


**Fig S1**. **Nanodrop 1000 spectrophotometry of cichlid tissue DNA extractions.** Mu-DNA-beads (red) and Mu-DNA-phenol:chloroform-beads (green) extraction methods. Vertical line indicates absorbance spectrum of nucleic acids (DNA/RNA).

It is clear the Mu-DNA-phenol:chloroform-beads method had higher DNA yields than Mu-DNA-beads (see Tables S1 and S2, Fig S1). However, given phenol extractions have the complications of requiring a fume hood and hazardous waste disposal, the Mu-DNA-Beads method was preferred. We determined Mu-DNA-Beads performed adequately for our purpose, yet the Mu-DNA-phenol:chloroform-beads is an optimal choice if applicable. Despite this both methods provided high yields of high purity DNA (Tables S1 and S2, Fig S1).

# Section 5: Library compositions

## RKN_lib2

RKN_lib2 raw fast5 sequencing data is available from SRA accession: SRR13976562 (not accessible via SRA toolkit).

12 samples: all *M. incognita*. Samples were collected and frozen ‘dry’ (i.e. not in molecular water). This is an ideal collection method for the extraction of DNA from RKN. Samples 1 to 8 were higher DNA yield while samples 9 to 12 were of low yield, with barcodes 9 and 12a being too low to quantify (Table S3).

**Table S3. Sample composition of library RKN_lib2.**
Sample id, species name, sample type (life stage of sample), sequencing barcode assigned to the sample, extracted DNA concentration and total DNA yield. Also shown is the SRA accession for the sample sequencing data.

| Sample id | Species | Sample type | Barcode | DNA conc (ng/µl) | DNA yield (ng) | SRA accession |
|---|---|---|---|---|---|---|
| mifi_13 | *M. incognita* | j4 or immature female | barcode01 | 0.105 | 2.625 | SRR13954828 |
| mifi_15 | *M. incognita* | j4 or immature female | barcode02 | 0.229 | 5.725 | SRR13954827 |
| mifi_16 | *M. incognita* | j4 or immature female | barcode03 | 0.112 | 2.8 | SRR13954824 |
| mifi_20 | *M. incognita* | j4 or immature female | barcode04 | 0.131 | 3.275 | SRR13954823 |
| mifi_22 | *M. incognita* | j4 or immature female | barcode05 | 0.113 | 2.825 | SRR13954822 |
| mifi_23 | *M. incognita* | j4 or immature female | barcode06 | 0.286 | 7.15 | SRR13954821 |
| mifi_24 | *M. incognita* | j4 or immature female | barcode07 | 0.167 | 4.175 | SRR13954820 |
| mifi_26 | *M. incognita* | j4 or immature female | barcode08 | 0.169 | 4.225 | SRR13954819 |
| mifi_14 | *M. incognita* | j4 or immature female | barcode09 | Too low | Too low | SRR13954818 |
| mifi_18 | *M. incognita* | j4 or immature female | barcode10 | 0.05 | 1.25 | SRR13954817 |
| mifi_19 | *M. incognita* | j4 or immature female | barcode11 | 0.053 | 1.325 | SRR13954826 |
| mifi_21 | *M. incognita* | j4 or immature female | barcode12a | Too low | Too low | SRR13954825 |

## RKN_lib3

RKN_lib3 raw fast5 sequencing data is available from SRA accession: SRR13977180 (not accessible via SRA toolkit).

10 samples: three *M. arenaria*, three *M. javanica*, three *M. hapla* and a single *M. incognita* (Table S4). All samples were of juvenile stage 4 (j4) or immature females. With the exception of *M. incognita*, samples were collected and stored in 50 µl molecular water. *M. incognita* was frozen ‘dry’ (i.e. not in molecular water). Due to complications during sample collection, these samples were left at room temperature for a prolonged period prior to freezing. This led to the death of the nematode and the proliferation of bacteria within the sample, resulting in fewer reads being assigned to *Meloidogyne*.

**Table S4. Sample composition of library RKN_lib3.**
Sample id, species name, sample type (life stage of sample), sequencing barcode assigned to the sample, extracted DNA concentration and total DNA yield. Also shown is the SRA accession for the sample sequencing data.

| Sample id | Species | Sample type | Barcode | DNA conc (ng/µl) | DNA yield (ng) | SRA accession |
|---|---|---|---|---|---|---|
| MA1 | *M. arenaria* | j4 or immature female | barcode01 | 0.347 | 8.675 | SRR13955590 |
| MA2 | *M. arenaria* | j4 or immature female | barcode02 | 0.36 | 9 | SRR13955589 |
| MA3 | *M. arenaria* | j4 or immature female | barcode03 | 0.366 | 9.15 | SRR13955588 |
| MH1 | *M. hapla* | j4 or immature female | barcode04 | 0.149 | 3.725 | SRR13955597 |
| MH2 | *M. hapla* | j4 or immature female | barcode05 | 0.488 | 12.2 | SRR13955596 |
| MH3 | *M. hapla* | j4 or immature female | barcode06 | 0.241 | 6.025 | SRR13955595 |
| MJ1 | *M. javanica* | j4 or immature female | barcode07 | 0.195 | 4.875 | SRR13955594 |
| MJ2 | *M. javanica* | j4 or immature female | barcode08 | 0.409 | 10.225 | SRR13955593 |
| MJ3 | *M. javanica* | j4 or immature female | barcode09 | 0.567 | 14.175 | SRR13955592 |
| MI1 | *M. incognita* | j4 or immature female | barcode10 | 0.564 | 14.1 | SRR13955591 |

## RKN_lib4

RKN_lib4 raw fast5 sequencing data is available from SRA accession: SRR13978602 (not accessible via SRA toolkit).

12 samples: three *M. enterolobii*, three *M. chitwoodi*, three *M. incognita* and three *M. hapla* (Table S5). All samples were of juvenile stage 4 (j4) or immature females with *M. hapla* being juvenile stage 2 (j2) individuals. All samples were frozen ‘dry’ (i.e. not in molecular water), *M. hapla* j2s were frozen in 50 µl molecular water. Due to complications during sample collection, *M. hapla* j2 samples were left at room temperature for a prolonged period prior to freezing. This led to the death of the j2s and the proliferation of bacteria within the sample, resulting in fewer reads being assigned to *Meloidogyne*. Storing j2s in molecular water caused issues: while pipetting off excess water, the sample can also be removed despite best efforts. This happened with MHJ1 (barcode 10).

**Table S5. Sample composition of library RKN_lib4.**
Sample id, species name, sample type (life stage of sample), sequencing barcode assigned to the sample, extracted DNA concentration and total DNA yield. Also shown is the SRA accession for the sample sequencing data.

| Sample id | Species | Sample type | Barcode | DNA conc (ng/µl) | DNA yield (ng) | SRA accession |
|---|---|---|---|---|---|---|
| ME1 | *M. enterolobii* | j4 or immature female | barcode01 | 0.37 | 9.25 | SRR13955570 |
| ME2 | *M. enterolobii* | j4 or immature female | barcode02 | 0.886 | 22.15 | SRR13955569 |
| ME3 | *M. enterolobii* | j4 or immature female | barcode03 | 0.656 | 16.4 | SRR13955566 |
| MC1 | *M. chitwoodi* | j4 or immature female | barcode04 | 0.824 | 20.6 | SRR13955565 |
| MC2 | *M. chitwoodi* | j4 or immature female | barcode05 | 0.442 | 11.05 | SRR13955564 |
| MC3 | *M. chitwoodi* | j4 or immature female | barcode06 | 0.48 | 12 | SRR13955563 |
| MFI1 | *M. incognita* | j4 or immature female | barcode07 | 0.686 | 17.15 | SRR13955562 |
| MFI2 | *M. incognita* | j4 or immature female | barcode08 | 1.07 | 26.75 | SRR13955561 |
| MFI3 | *M. incognita* | j4 or immature female | barcode09 | 0.77 | 19.25 | SRR13955560 |
| MHJ1 | *M. hapla* | j2 | barcode10 | Too low | Too low | SRR13955559 |
| MHJ2 | *M. hapla* | j2 | barcode11 | Too low | Too low | SRR13955568 |
| MHJ3 | *M. hapla* | j2 | barcode12a | Too low | Too low | SRR13955567 |

## RKN_lib5

RKN_lib5 raw fast5 sequencing data is available from SRA accession: SRR14431496 (not accessible via SRA toolkit).

12 samples: three *M. arenaria*, three *M. incognita,* three *M. javanica* and three *M. hapla* (Table S6). All samples were of juvenile stage 2 (j2). All samples were frozen 'dry' (i.e. not in molecular water). Two *M. javanica* samples failed to sequence successfully, MJJ2 (barcode08) and MJJ3 (barcode09), possibly due to loss of sample in the tube or failure to transfer sufficient DNA template for library preparation.

**Table S6. Sample composition of library RKN_lib5.**
Sample id, species name, sample type (life stage of sample), sequencing barcode assigned to the sample, extracted DNA concentration and total DNA yield. Also shown is the SRA accession for the sample sequencing data.

| Sample id | Species | Sample type | Barcode | DNA conc (ng/µl) | DNA yield (ng) | SRA accession |
|---|---|---|---|---|---|---|
| MAJ1 | *M. arenaria* | j2 | barcode01 | Too low | Too low | SRR14428432 |
| MAJ2 | *M. arenaria* | j2 | barcode02 | Too low | Too low | SRR14428431 |
| MAJ3 | *M. arenaria* | j2 | barcode03 | Too low | Too low | SRR14428428 |
| MIJ1 | *M. incognita* | j2 | barcode04 | Too low | Too low | SRR14428427 |
| MIJ2 | *M. incognita* | j2 | barcode05 | Too low | Too low | SRR14428426 |
| MIJ3 | *M. incognita* | j2 | barcode06 | Too low | Too low | SRR14428425 |
| MJJ1 | *M. javanica* | j2 | barcode07 | Too low | Too low | SRR14428424 |
| MJJ2 | *M. javanica* | j2 | barcode08 | Too low | Too low | SRR14428423 |
| MJJ3 | *M. javanica* | j2 | barcode09 | Too low | Too low | SRR14428422 |
| MHJ4 | *M. hapla* | j2 | barcode10 | Too low | Too low | SRR14428421 |
| MHJ5 | *M. hapla* | j2 | barcode11 | Too low | Too low | SRR14428430 |
| MHJ6 | *M. hapla* | j2 | barcode12a | Too low | Too low | SRR14428429 |

## RKN_lib6

RKN_lib6 raw fast5 sequencing data is available from SRA accession: SRR14434158 (not accessible via SRA toolkit).

12 mixed species samples (Table S7). All samples were of two species with a single juvenile stage 2 (j2) individual of each. All samples were frozen 'dry' (i.e. not in molecular water).

**Table S7. Sample composition of library RKN_lib6.**
Sample id, species names, sample type (life stage of sample), sequencing barcode assigned to the sample, extracted DNA concentration and total DNA yield. Also shown is the SRA accession for the sample sequencing data.

| Sample id | Species | Sample type | Barcode | DNA conc (ng/µl) | DNA yield (ng) | SRA accession |
|---|---|---|---|---|---|---|
| MJMHJ1 | *M. javanica* and *M. hapla* | j2 | barcode01 | Too low | Too low | SRR14432434 |
| MJMHJ2 | *M. javanica* and *M. hapla* | j2 | barcode02 | Too low | Too low | SRR14432433 |
| MJMHJ3 | *M. javanica* and *M. hapla* | j2 | barcode03 | Too low | Too low | SRR14432430 |
| MIMAJ1 | *M. incognita* and *M. arenaria* | j2 | barcode04 | Too low | Too low | SRR14432429 |
| MIMAJ2 | *M. incognita* and *M. arenaria* | j2 | barcode05 | Too low | Too low | SRR14432428 |
| MIMAJ3 | *M. incognita* and *M. arenaria* | j2 | barcode06 | Too low | Too low | SRR14432427 |
| MIMJJ1 | *M. incognita* and *M. javanica* | j2 | barcode07 | Too low | Too low | SRR14432426 |
| MIMJJ2 | *M. incognita* and *M. javanica* | j2 | barcode08 | Too low | Too low | SRR14432425 |
| MIMJJ3 | *M. incognita* and *M. javanica* | j2 | barcode09 | Too low | Too low | SRR14432424 |
| MJMAJ1 | *M. javanica* and *M. arenaria* | j2 | barcode10 | Too low | Too low | SRR14432423 |
| MJMAJ2 | *M. javanica* and *M. arenaria* | j2 | barcode11 | Too low | Too low | SRR14432432 |
| MJMAJ3 | *M. javanica* and *M. arenaria* | j2 | barcode12a | Too low | Too low | SRR14432431 |

# Section 6: Database cleanup

RKN reference genomes were cleaned of contaminants. The number of contaminated contigs removed from each reference genome (those assigned to bacteria, human and plant) are shown in Table S8.

**Table S8. RKN genomes used for database creation.**
Species name, accession numbers and contig counts per genome used in our reference database. The number of contigs assigned to bacteria and contaminants (human and plant) are shown along with the remaining number of contigs after the removal of contigs considered contaminated.

| Species name | Accession number | Contigs | Contigs assigned to bacteria | Contigs assigned to contaminants | Contigs remaining |
|---|---|---|---|---|---|
| *Meloidogyne arenaria* | GCA_003133805.1 | 2224 | 0 | 7 | 2217 |
| *Meloidogyne arenaria* | GCA_003693565.1 | 46436 | 5 | 15 | 46416 |
| *Meloidogyne arenaria* | GCA_900003985.1 | 26196 | 3 | 30 | 26163 |
| *Meloidogyne chitwoodi* | GCA_015183025.1 | 38 | 0 | 0 | 38 |
| *Meloidogyne chitwoodi* | GCA_015183035.1 | 30 | 0 | 0 | 30 |
| *Meloidogyne chitwoodi* | GCA_015183065.1 | 39 | 0 | 0 | 39 |
| *Meloidogyne enterolobii* | GCA_003693675.1 | 42008 | 5 | 18 | 41985 |
| *Meloidogyne enterolobii* | GCA_903797545.1 | 4437 | 0 | 17 | 4420 |
| *Meloidogyne enterolobii* | GCA_903994135.1 | 4437 | 0 | 17 | 4420 |
| *Meloidogyne floridensis* | GCA_000751915.1 | 58696 | 89 | 15 | 58592 |
| *Meloidogyne floridensis* | GCA_003693605.1 | 8887 | 0 | 1 | 8886 |
| *Meloidogyne graminicola* | GCA_002778205.1 | 4304 | 7 | 2 | 4295 |
| *Meloidogyne graminicola* | GCA_014773135.1 | 283 | 0 | 2 | 281 |
| *Meloidogyne hapla* | GCA_000172435.1 | 3450 | 37 | 6 | 3407 |
| *Meloidogyne incognita* | GCA_000180415.1 | 9538 | 0 | 6 | 9532 |
| *Meloidogyne incognita* | GCA_003693645.1 | 33351 | 2 | 11 | 33338 |
| *Meloidogyne incognita* | GCA_014132215.1 | 374 | 0 | 3 | 371 |
| *Meloidogyne incognita* | GCA_900182535.1 | 12091 | 2 | 38 | 12051 |
| *Meloidogyne javanica* | GCA_003693625.1 | 34316 | 9 | 9 | 34298 |
| *Meloidogyne javanica* | GCA_900003945.1 | 31341 | 1 | 22 | 31318 |
| *Meloidogyne luci* | GCA_902706615.1 | 327 | 0 | 1 | 326 |

# Section 7: Method time and cost

Dna extraction, library prep, sequencing and analysis time calculations were based upon a familiar or expert user of the method (Table S9). Flongle sequencing cost estimates, including consumables, were based on current pricing of relevant products and reagents used (Table S10).

**Table S9. Time calculations for completion for each process of the method.**
Times for completion and staff hands-on time (staff time) per process based on a user familiar with the method. More complex processes (e.g. DNA extraction and library preparation) have times per step shown (italicised) in addition to the total and staff times for the process (bold).

| Process | Time to complete | Staff time |
|---|---|---|
| **DNA extraction** | **2 hrs 55 mins** | **1 hr** |
| *Preparation* | *20 mins* | *20 mins* |
| *Lysis* | *1 hr 30 mins* | *5 mins* |
| *Inhibitor removal* | *20 mins* | *10 mins* |
| *SPRI binding* | *20 mins* | *10 mins* |
| *Wash* | *10 mins* | *10 mins* |
| *Elution* | *15 mins* | *5 mins* |
| **Library preparation** | **5 hrs 15 mins** | **2 hrs 10 mins** |
| *preparation* | *20 mins* | *20 mins* |
| *DNA fragmentation* | *10 mins* | *10 mins* |
| *PCR prep* | *10 mins* | *10 mins* |
| *PCR* | *2 hrs* | *5 mins* |
| *Concentration* | *30 mins* | *10 mins* |
| *DNA quantification* | *10 mins* | *10 mins* |
| *Fragment length estimation* | *1 hr* | *20 mins* |
| *Pool samples* | *10 mins* | *10 mins* |
| *Adapter ligation* | *15 mins* | *5 mins* |
| *Loading Flongle* | *30 mins* | *30 mins* |
| **Sequencing** | **24 hrs** | **0 mins** |
| **Guppy basecalling** | **40 mins** | **5 mins** |
| **Analysis** | **10 mins** | **5 mins** |
| **Total time** | **33 hrs** | **3 hrs 20 mins** |

**Table S10. Costing per sample for sequencing 12 samples on a single ONT Flongle flow cell following our method.**
Current costs in GBP per item (excluding VAT and shipping) and calculated as total cost per sample sequenced. The supplier and catalogue number is provided per item.

| **Item/reagent** | **Supplier** | **Cost (GBP)** | **Pack size/ volume (µl)/ reactions** | **Cost per piece** | **Used per sample** | **Cost per sample** |
|---|---|---|---|---|---|---|
| Flongle flow cell | ONT FLO-FLG001 | 73.00 | 1 | 73.00 | 0.08 | 6.08 |
| Sequencing kit | ONT SQK-RPB004 | 520.00 | 72 | 7.22 | 1.00 | 7.22 |
| Eppendorf 1.5ml LoBind tubes | Fisher Scientific 10051232 | 15.07 | 250 | 0.06 | 4.00 | 0.24 |
| 1000 ul filter tips | Starlab UK S1122-1730 | 75.14 | 960 | 0.08 | 8.00 | 0.63 |
| 200 ul filter tips | Starlab UK S1120-8710 | 71.23 | 960 | 0.07 | 6.00 | 0.45 |
| 100 ul filter tips | Starlab UK S1123-1840 | 79.12 | 960 | 0.08 | 3.00 | 0.25 |
| 10 ul filter tips | Starlab UK S1121-2710 | 75.14 | 960 | 0.08 | 6.00 | 0.47 |
| PCR tubes | Starlab UK I1402-3700 | 128.04 | 960 | 0.13 | 5.00 | 0.67 |
| LongAmp Taq 2X Master Mix | NEB UK M0287S | 144 | 2500 | 0.06 | 25.00 | 1.44 |
| Qubit hsDNA assay | ThermoFisher Q32854 | 211 | 500 | 0.42 | 1.00 | 0.42 |
| | | | | | **Total cost per sample (GBP)** | **17.86** |
